# ZnO Hierarchical Nanostructure Photoanode in a CdS Quantum Dot-Sensitized Solar Cell

**DOI:** 10.1371/journal.pone.0138298

**Published:** 2015-09-17

**Authors:** Huan Liu, Gengmin Zhang, Wentao Sun, Ziyong Shen, Mingji Shi

**Affiliations:** 1 Key Laboratory for the Physics and Chemistry of Nanodevices and Department of Electronics, Peking University, Beijing, China; 2 SIP-UCLA Institute for Technology Advancement, Suzhou, Jiangsu Province, China; 3 School of Electronic and Electrical Engineering, Nanyang Institute of Technology, Nanyang, Henan Province, China; Institute for Materials Science, GERMANY

## Abstract

A hierarchical array of ZnO nanocones covered with ZnO nanospikes was hydrothermally fabricated and employed as the photoanode in a CdS quantum dot-sensitized solar cell (QDSSC). This QDSSC outperformed the QDSSC based on a simple ZnO nanocone photoanode in all the four principal photovoltaic parameters. Using the hierarchical photoanode dramatically increased the short circuit current density and also slightly raised the open circuit voltage and the fill factor. As a result, the conversion efficiency of the QDSSC based on the hierarchical photoanode was more than twice that of the QDSSC based on the simple ZnO nanocone photoanode. This improvement is attributable to both the enlarged specific area of the photoanode and the reduction in the recombination of the photoexcited electrons.

## Introduction

The key components of a sensitized solar cell are the photoanode, sensitizer (dye molecules or semiconductor quantum dots) and electrolyte. The photoanode both loads the sensitizers and offers channels for the transportation of photogenerated carriers. Metal oxides play an important role in optoelectronics [1,2]. A variety of metal oxides, e.g., titanium dioxide (titania, TiO_2_), zinc oxide (ZnO) and tungsten oxide (WO_3_), can be used as the photoanodes [3–5]. So far, TiO_2_ is still by far the most suitable semiconductor material for the photoanodes. Due to a variety of appealing physical and chemical properties, ZnO has demonstrated application perspectives in many areas[6–13]. Importantly, it is also considered to be a possible alternative to TiO_2_ as the photoanode material in sensitized solar cells[14–22]. The bulk electron mobility of ZnO is more than one order of magnitude larger than that of TiO_2_ and this better electron transport property may alleviate the mass transport limitations in the solar cells [3,23,24]. Moreover, among all the metal oxides, ZnO also has the richest family of nanostructures, so that the morphology of the photoanode might be more controllable. Especially, in some quantum-dot-sensitized solar cells (QDSSCs), photoanodes based on one-dimensional (1D) ZnO nanostructures, such as ZnO nanorods and ZnO nanowires, are employed to load the QDs and provide the transportation channels for the photoexcited electrons. In comparison with semiconductor nanoparticles, there are relatively fewer grain boundaries in these 1D ZnO nanostructures, thus charge recombination can be suppressed to a certain degree [25–27]. Nonetheless, the capability of attaching QDs and harvesting incident photons of these 1D ZnO nanostructures is limited by their low specific areas. Similar problem also exists in the 1D nanostructure-based photoanodes in the dye-sensitized solar cells (DSSCs). For overcoming this difficulty, hierarchical structured photoanodes have been developed in both DSSCs and QDSSCs [28–47].

Among these works, relatively less effort has been devoted to the QDSSCs than to the DSSCs. In this context, a ZnO hierarchical photoanode with a different configuration from the previous ones has been developed in this lab. The CdS QD-sensitized ZnO photoanodes are usually synthesized by the initial growth of the ZnO nanostructures and the subsequent deposition of the CdS QDs [48–53]. In this work, cone-shaped primary nanostructures were used for a large space between them to accommodate sufficient electrolyte. Spike-shaped secondary nanostructures were used for a specific area as large as possible. Both the primary and the secondary nanostructures were ZnO. These ZnO hierarchical nanostructures were grown using a facile and inexpensive hydrothermal method.

## Experimental Section

Each ZnO hierarchical nanostructure array was fabricated in two steps, namely the growth of the primary ZnO nanostructure array and the subsequent growth of the secondary ZnO branches on these primary nanostructures. The primary ZnO nanostructure array was grown on a fluorine-doped tin oxide (FTO) glass substrate using a field-assisted method, whose details can be found in Ref.[54]. The precursor was an aqueous solution that contained 0.02 M zinc nitrate (Zn(NO_3_)_2_) and 0.02 M hexamethylenetetramine (HMTA, C_6_H_12_N_4_). A beaker that contained this solution was immersed in a water bath and two electrodes were inserted into the solution. The FTO substrate was used as the cathode and a Pt wire was used as the anode. The reaction occurred under 90°C with a 2.5 V voltage applied to the two electrodes. The reaction time was controlled to be 3, 6 or 9 h. Then the FTO substrate with the primary ZnO nanostructures was immersed in a limpid aqueous solution of 0.057 M zinc acetate (Zn(CH_3_COO)_2_·2H_2_O, ZnAc) and 0.5 M sodium hydroxide (NaOH) under constant stirring at room temperature for the growth of the secondary ZnO branches, so that a hierarchical ZnO nanostructure array was finally obtained [34]. (It is worth stressing that the limpid solution would become turbid about 5 min after NaOH and ZnAc were dissolved and secondary ZnO nanostructures would not be available if the FTO substrate was inserted in a turbid solution. That is, for obtaining the secondary ZnO nanostructures, the FTO substrate should be inserted to the solution right after the NaOH and ZnAc were dissolved in water.) After this, the sample was repeatedly rinsed using deionized water and ethanol.

For the deposition of CdS QDs[48,55], the FTO substrate was further immersed in a mixed solution of 5 mM cadmium nitrate (Cd(NO_3_)_2_) and 5 mM thioacetamide (C_2_H_5_NS) in 100 mL deionized water for 30 min, and then rinsed with deionized water and ethanol. Finally the sample was annealed in air at 400°C for 30 min.

The ZnO hierarchical nanostructure arrays, either with or without the CdS QDs on them, were characterized using such means as scanning electron microscopy (SEM, FEI Quanta 600 microscopy), transmission electron microscopy and energy dispersive X-ray spectroscopy (TEM and EDS, JEM-2100F), X-ray diffraction (XRD, DMAX-2400) and UV-vis spectrophotometry (UV 5000 spectrometers, Gary). For the TEM observation, part of the ZnO nanostructures were scratched down from the photoanodes and dispersed in ethanol. Then some ethanol drops with the ZnO nanostructures were applied to the C thin films on the Cu meshes.

For assembling a QDSSC, the CdS sensitized ZnO electrode and a platinized FTO counter electrode were sealed together with a 60-μm-thick hot-melt surlyn spacer. The ZnO nanostructures loaded with CdS QDs were thus sandwiched between two transparent FTO electrodes. An I^−^/I_3_^−^ based electrolyte (DHS-E23, Dalian HeptaChromaSolarTech, China) was then injected into the space between the two electrodes through holes in the counter electrode.

Photovoltaic properties of the QDSSCs were measured under AM 1.5 simulated sunlight at 100 mW·cm^−2^ (Oriel Solar Simulator, Model 91160). The exposed area was 0.25 cm^2^. First, the dependence of the photocurrent density on the photovoltage (*J*-*V* curves) was recorded. Then, the incident-photon-to-current efficiency (IPCE) spectra were analyzed in the wavelength range from 350 to 800 nm. Finally, more properties of the cells were disclosed using the electrochemical impedance spectroscopy (EIS). The EIS measurements were performed at the V_OC_s under dark conditions.

## Results and Discussion

**Fig 1:**
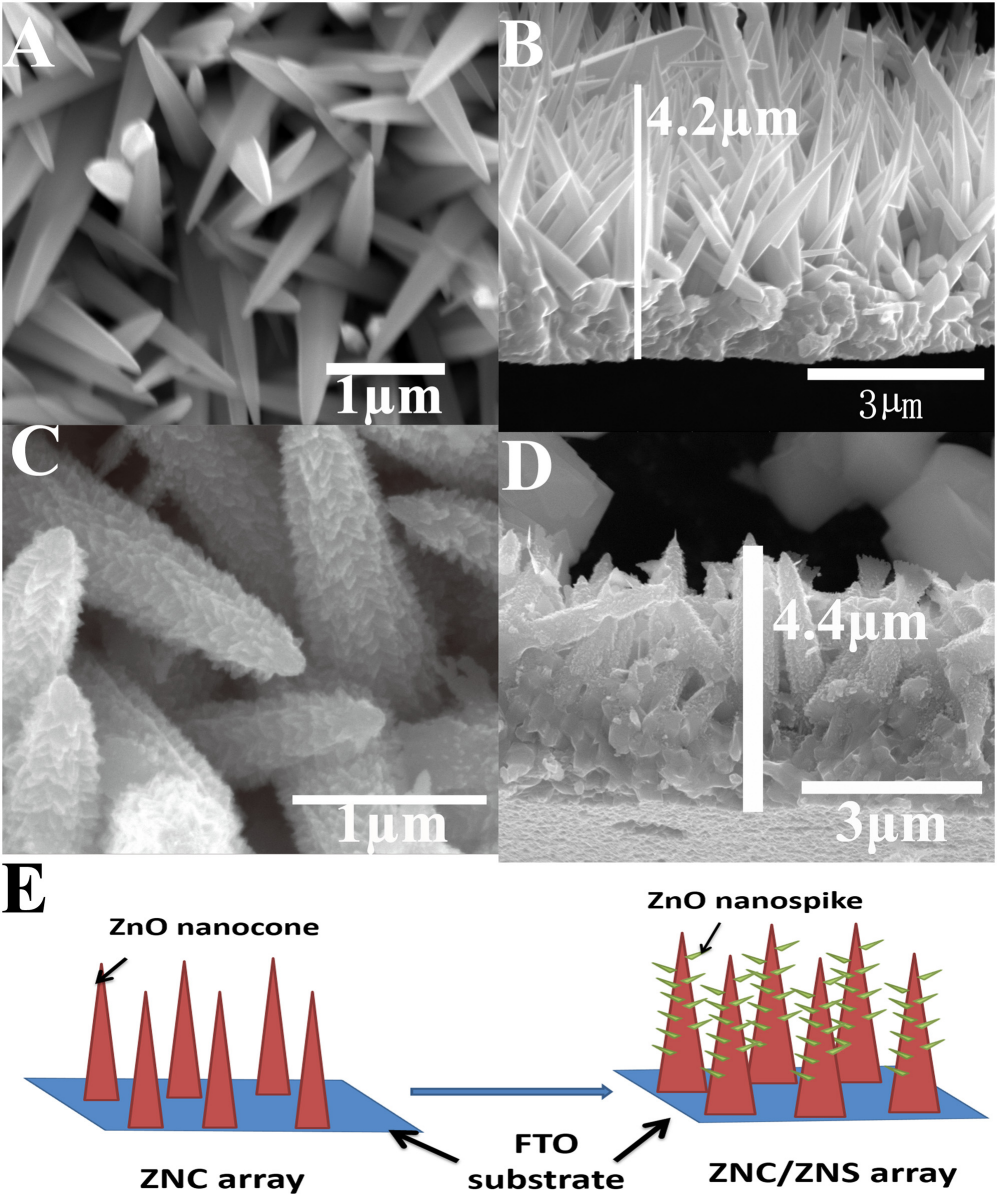
SEM images of the ZNC(3) arrays and the hierarchical ZNC(3)/ZNS arrays. (A) top view and (B) side view of a ZNC(3) array, (C) top view and (D) side view of a ZNC(3)/ZNS array, and (E) the schematic of the two-step growth process of a ZNC/ZNS array.

The cone-shaped primary ZnO nanostructures shown in Fig 1A and 1B, which were fabricated in the aqueous solution of Zn(NO_3_)_2_ and HMTA, are all roughly upwards aligned. Hereafter these ZnO nanostructures are referred to as “ZnO nanocones (ZNC(x)s)”, where “x” denotes the growth time in hour. As introduced in the “Experimental” section, the value of “x” can be 3, 6 or 9. As shown in Fig 1A and 1B, the ZnO nanocones grown in 3 h (ZNC(3)s) were around 4.2 μm in height. When the growth time was prolonged to 6 and 9 h, the height increased to 5.7 and 6.6 μm, respectively. (S1 Fig) After the reaction in the aqueous solution of ZnAc and NaOH, as shown in Fig 1C and 1D, the previously smooth ZNC surfaces were covered with secondary small protrusions, hereafter referred to as “ZnO nanospikes (ZNSs)”, and became quite rough. The height of the ZNC(3)/ZNS, ZNC(6)/ZNS and ZNC(9)/ZNS arrays increased only slightly to 4.4 (Fig 1C), 5.8 and 6.9 μm (S1 Fig), respectively, and the thickening was also detectable but not considerable. The process of the two-step growth of such a ZNC/ZNS hierarchical nanostructure array is shown in Fig 1E.

**Fig 2:**
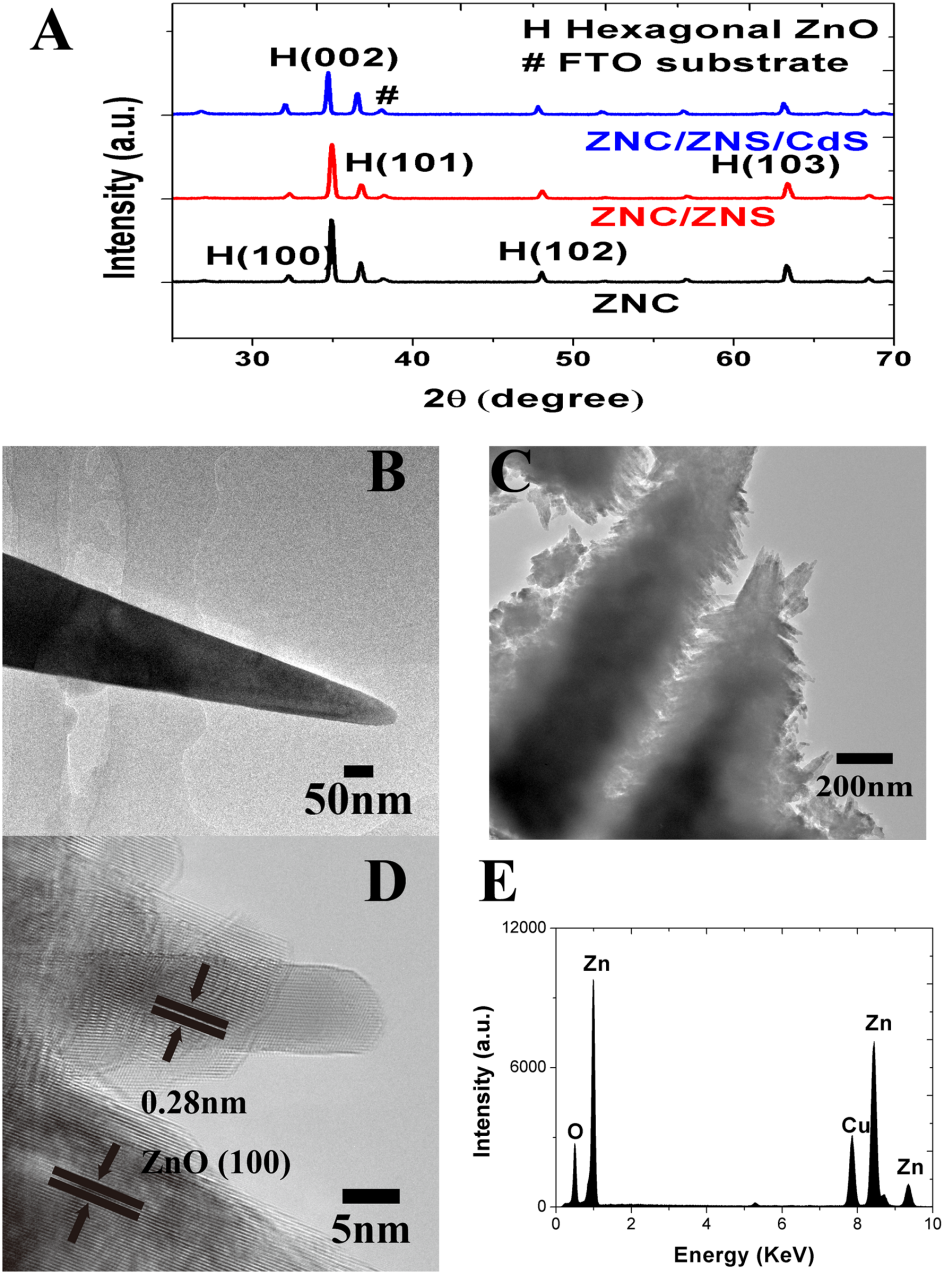
The XRD patterns and TEM images of the ZNCs and ZNC/ZNS nanostructures. (A) XRD patterns of a ZNC array, a ZNC/ZNS array and a CdS QD-sensitized ZNC/ZNS array, (B) TEM image of a ZNC, (C) TEM image and (D) HRTEM image of a ZNC/ZNS nanostructure, (E) EDS of a ZNC/ZNS nanostructure.

The results of the XRD analysis on a ZNC array, a ZNC/ZNS array and a CdS QD-sensitized ZNC/ZNS array are shown in Fig 2A together and little difference is observable between them, demonstrating that the crystal structure of the primary ZNC and the secondary ZNS were similar to each other. The main peaks at 2θ values of 32.2, 34.9, 36.7, 48.0, and 63.3° can be respectively indexed to the (100), (002), (101), (102) and (103) crystal planes of the hexagonal phase ZnO (JCPDS No. 36–1451). The CdS QDs were too small to be detected in the XRD analysis. The TEM images given in Fig 2B and 2C further disclosed the smooth surface of a ZNC and the surface decorated with small protrusions of a ZNC/ZNS nanostructure. These results obtained from individual nanostructures are in agreement with those obtained from arrays shown in Fig 1. In the HRTEM image shown in Fig 2D, the crystal planes are separated by 0.28 nm, which is in accordance with the interplanar spacing of the (100) crystal planes of the wurtzite ZnO (JCPDS No. 36–1451). In the EDS shown in Fig 2E, except for the Cu peak arising from the supporting mesh, only the Zn and O elements are detectable. This result shows that the residuals on the samples during the reaction, e.g., elemental sodium, were all successfully removed by the rinsing and the purity of the samples could be guaranteed.

As previously reported by this lab [54,56], on the one hand, the ZnO growth could occur even without the application of an external voltage; on the other hand, the application of an external voltage indeed greatly improved the orderliness of the ZnO nanostructures. Therefore, both a hydrothermal growth mechanism and an electrochemical growth mechanism were likely to have contributed to the growth of the ZNCs in this work.

As a common knowledge, Zn^2+^ cations occur largely in four-coordination as tetrahedral complexes [57]. Thus it is believed that Zn(OH)_4_^2-^s were the precursors of the ZnO growth in this work. That is, the formation of Zn(OH)_4_^2–^ precursors and their subsequent incorporation into the ZnO crystals resulted in the growth of the ZnO crystals [58]. Obviously, the Zn^2+^s in these precursors came from the Zn(NO_3_)_2_. The existence of the hydroxide anions (OH^-^s) is attributed to the use of the HMTA. The HMTA slowly decomposed to produce ammonia (NH_3_) in a gradual and controlled manner, which could form ammonium hydroxide (NH_4_OH) and provide the OH^-^s [59,60]. The reactions in the solution can be simplified as described by the following formulae[61–63]:

$${\left({\text{CH}}_{\text{2}}\right)}_{\text{6}}{\text{N}}_{\text{4}}{\text{+6H}}_{\text{2}}\text{O}\to {\text{6HCHO+4NH}}_{\text{3}}$$ (1)

$${\text{NH}}_{\text{3}}{+\text{ H}}_{\text{2}}\text{O}\to {\text{NH}}_{\text{4}}{}^{\text{+}}{+\text{ OH}}^{-}$$ (2)

$${\text{Zn}}^{\text{2+}}{\text{+4OH}}^{-}\to \text{Zn}{\left(\text{OH}\right)}_{\text{4}}{}^{2-}$$ (3)

$$\text{Zn}{\left(\text{OH}\right)}_{\text{4}}{}^{2-}\to {\text{ZnO+H}}_{\text{2}}{\text{O+2OH}}^{-}$$ (4)

In a ZnO crystal, the Zn-terminated (0001) plane had a high surface energy, thus Zn(OH)_4_^2–^ precursors were preferentially adsorbed to the (0001) plane [64]. Moreover, besides providing the OH^-^s, the HMTA also played an important role in hindering the growth of some planes [65]. For example, as proposed by Sugunan et al., HMTA was preferentially attached to some nonpolar planes of the ZnO crystal and thus cut off the access of Zn^2+^s to these planes[66]. Consequently, the c-axis became the major growth direction.

The application of an external voltage presumably triggered the following electrochemical reactions [67–69]:

$${\text{NO}}_{\text{3}}{}^{-}{\text{+H}}_{\text{2}}{\text{O+2e}}^{-}\to {\text{NO}}_{\text{2}}{}^{-}{\text{+2OH}}^{-}$$ (5)

$${\text{NO}}_{\text{3}}{}^{-}{\text{+6H}}_{\text{2}}{\text{O+8e}}^{-}\to {\text{NH}}_{\text{3}}{\text{+9OH}}^{-}$$ (6)

Hence, besides reactions (1) and (2), the electrochemical reactions described in (5) and (6) also generated OH^-^s in the solution, providing more reactants for reactions (3) and (4). With excessive OH^-^s, the movement of the Zn^2+^s to the ZnO crystal was likely to become the rate-determining step in the ZnO growth. Tena-Zaera et al argued that ZnO nanowires mainly grew along the longitudinal axis if the diffusion of Zn^2+^s was slower than the generation of OH^-^s [70]. Under the negative electric field at the cathode surface, on the one hand, more Zn^2+^s moved to the vicinity of the ZnO crystals; on the other hand, the negatively charged Zn(OH)_4_^2–^ precursors generated in reaction (3) received a repulsion from the cathode and became more difficult to be adsorbed onto the ZnO crystal planes. As a result, only the growth along the c-axis continued due to the high reactivity of the (0001) plane [64,71]. The eventual result was that the negative electric field enhanced the growth along the c-axis by attracting more Zn^2+^s and hindered the growth along other directions by repulsing the Zn(OH)_4_^2–^ precursors.

Electric field around a sharp end of a ZnO crystal was stronger than that around a flat end, thus a sharp end could attract more Zn^2+^ s and grew faster. Therefore, the primary ZnO nanostructures in this work became cone-shaped under the external electric fields. Moreover, it is possible that the erosion by the OH^-^s around the boundaries of the (0001) planes also contributed to the tapering of the ZnO nanostructures [72].

The crucial factor for the growth of the secondary ZNSs was the formation of the “etch pits” on the surfaces of the primary ZNCs. From these etch pits, the nanospikes, which were much smaller than the nanocones, were further developed in the supersaturate aqueous solutions of ZnAc and NaOH [34]. When an FTO substrate with ZNCs on it was immersed in the limpid solution with high concentration NaOH, the OH^-^s could erode the already existent ZnO nanostructures [72]:

$${\text{ZnO }+\;2\text{OH}}^{-}\to {\text{ZnO}}_{\text{2}}{}^{2-}{+\text{ H}}_{\text{2}}\text{O}$$ (7)

A large number of etch pits resulted from this reaction on the ZNC surfaces and constituted the starting places for the growth of the ZNSs.

According to the analysis in Refs. [73–75], the Zn(OH)_4_^2–^ precursors still played a vitally important role in the growth of the secondary ZNSs, whose process can as well be described by formulae (3) and (4). This time the Zn^2+^ cations and the OH^-^anions came from the ZnAc and NaOH, respectively. In comparison with some methods of growing ZnO nanowires and nanoforests, the growth of the nanocones and the nanospikes in this work, which did not involve any organic structure-directing agents, seeding process and heating process, appeared to be simpler and less costly.

**Fig 3:**
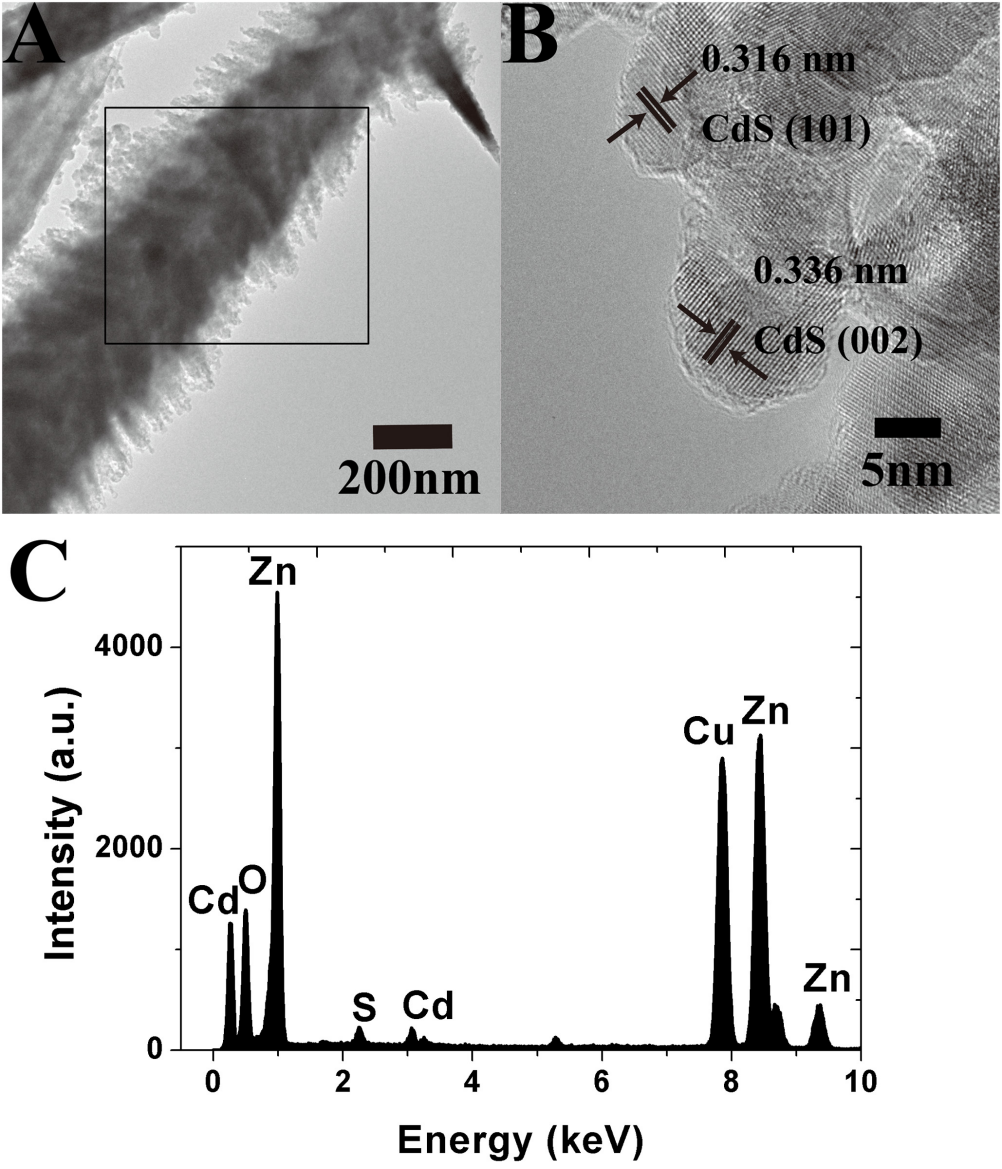
ZNC/ZNS nanostructures loaded with CdS QDs. (A) a TEM image, (B) an HRTEM image, and (C) EDS.

Fig 3A and 3B show the TEM and HRTEM images of the ZNC/ZNS nanostructures with some QDs, ~6 nm in size, adsorbed on them. Fig 3B also shows lattice fringes of 0.316 and 0.336 nm, which can be indexed as the (101) and (002) planes of CdS (JCPDS 10–0454). The selected area EDS of the sample, shown in Fig 3C, further confirms the elementary composition of the CdS-coated nanostructures, which, as expected, are Zn, O, Cd and S elements. (The Cu peak arose from the supporting Cu mesh in the TEM observation.)

**Fig 4:**
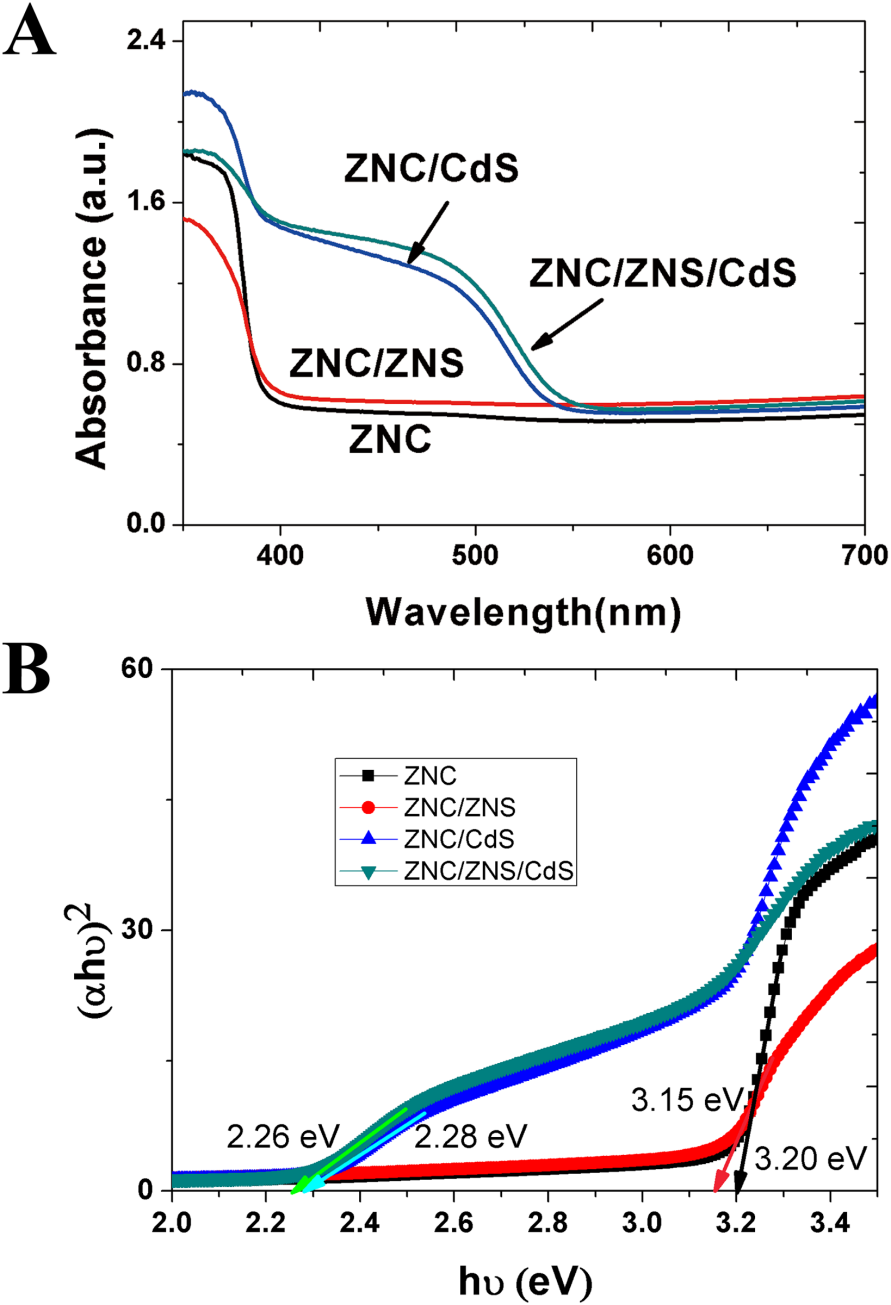
UV–vis absorption spectra and band gap estimation of ZnO and ZnO/CdS nanostructures. (A) UV–vis absorption spectra; (B) band gap estimation.

The UV-vis absorption spectra of the as prepared ZnO nanostructures and the CdS sensitized ZnO nanostructures, which were calculated from the reflectance spectra and the transmission spectra (S2 Fig), are given in Fig 4A.

The energy band gaps of the ZnO nanostructures and CdS QDs were estimated using the formula [76]:

$$\alpha \cdot h\nu =A{\left(h\nu -E_{g}\right)}^{n},$$ (8)

where *A* is a constant, *α* the absorption coefficient, *hν* the photon energy and *E*_*g*_ the energy band gap. The value of *n* depends on the type of the semiconductor. Since both CdS and ZnO are direct semiconductors, *n* is 0.5 [76–78]. As shown in Fig 4B, the linear parts of the dramatic increase in (*α*·*hν*)^2^ were extrapolated to the low photon energy end and the intersections with the abscissa are considered to be the band gap values. Using this method, the band gaps of the ZnO nanostructures and CdS QDs are estimated to be 3.2 and 2.3 eV, respectively. These results are in good agreement with the values given in Ref. [79], which reported the band gaps of CdS and ZnO to be 2.25 and 3.2 eV, respectively.

As shown in Fig 4, no matter the nanostructures were covered with the CdS QDs or not, the visible light absorption of the hierarchical ZNC/ZNS nanostructures was stronger than that of the ZNCs, indicating the use of the hierarchical nanostructures could improve the light harvesting efficiency of the photoanode.

**Fig 5:**
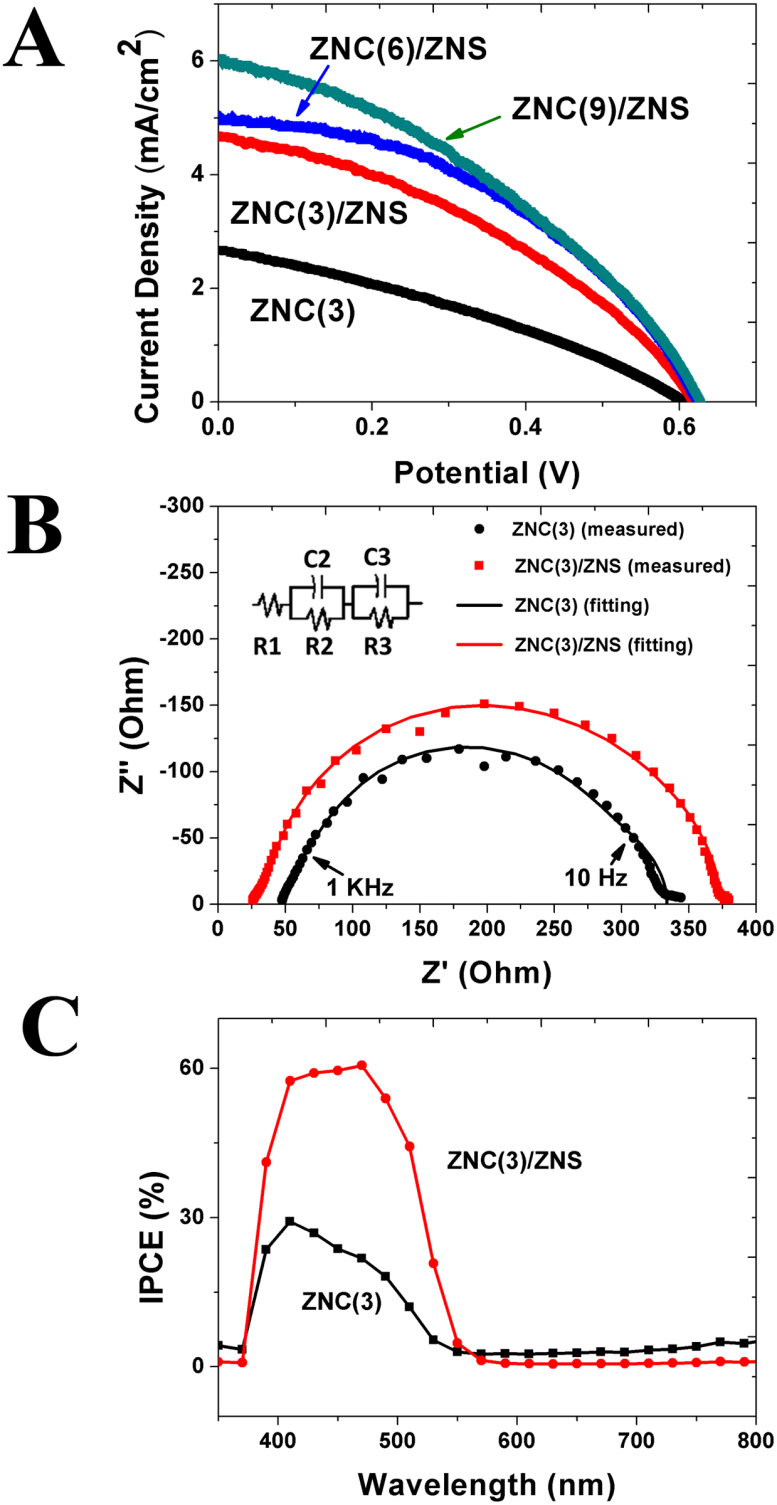
Photovoltaic properties of the CdS-sensitized cells with the ZNC and ZNC/ZNS nanostructures as the photoanodes. (A) *J*-*V* curves, (B) Nyquist plots (The equivalent circuit is given as the inset. The dots are experimental data and the two semicircle lines are the results of fitting.) (C) IPCE curves.

The ZNC and ZNC/ZNS nanostructure arrays were loaded with CdS QDs and then respectively used as the photoanodes of the solar cells. Their *J*-*V* curves, EIS and IPCE curves are shown in Fig 5. The short-circuit current density (*J*_*SC*_), open-circuit voltage (*V*_*OC*_), fill factor (*FF*) and conversion efficiency (*η*) of the cells are listed in Table 1. The values of the parameters that describe the properties of the ZnO/CdS/electrolyte interfaces are given in Table 2. The *η* of the cell based on the ZNC(3)/ZNS photoanode is more than twice that of the cell based on the ZNC(3) photoanode (1.1% vs. 0.53%). This improvement mainly arises from a 76% increase in the *J*_SC_ of the ZNC(3)/ZNS-based cell over that of the ZNC(3)-based cell (4.67 vs. 2.66 mA·cm^−2^). In total, 20 ZNC(3)/ZNS-based QDSSCs were fabricated and their photovoltaic properties were quite reproducible (S3 Fig). The hierarchical nanostructures of the ZNC/ZNS photoanode accommodated more CdS QDs and also trapped more incident light inside the photoanode, thus a larger *J*_SC_ resulted.

**Table 1:** Photovoltaic properties of the CdS-sensitized cells with the ZNC photoanodes and the ZNC/ZNS photoanodes.

Photoanode	*J*_*SC*_ (mA cm^−2^)	*V*_*OC*_ (V)	*FF*	*η*(%)
**ZNC(3)**	2.66	0.605	0.33	0.53
**ZNC(3)/ZNS**	4.67	0.618	0.37	1.1
**ZNC(6)/ZNS**	5.00	0.624	0.42	1.3
**ZNC(9)/ZNS**	6.06	0.625	0.37	1.4

**Table 2:** Values of the resistance and capacitance per unit area across the photoanodes at the ZnO/CdS/electrolyte interfaces obtained from the fitting of the data shown in Fig 5B.

Anode	*R*_3_ (kΩ cm^2^)	*C*_3_ (μF cm^−2^)	*R*_3_·*C*_3_ (ms)
**ZNC(3)**	0.22	4.3	0.9
**ZNC(3)/ZNS**	0.24	5.9	1.4

The results given in Fig 5A and Table 1 show that prolonging the ZNC length was effective in raising the conversion efficiency. The conversion efficiencies of the cells based on ZNC(6)/ZNS and ZNC(9)/ZNS were 1.3% and 1.4%, which were 18% and 27% higher than that of the ZNC(3)/ZNS-based cell, respectively. Table 1 further shows that this improvement in the conversion efficiency mainly arose from the increase in *J*_SC_ (4.67 vs. 5.00 & 6.06 mA cm^−2^). The actual surface areas of the photoanodes were enlarged by prolonging the ZNCs, thus more QDs were adsorbed on them and larger *J*_SC_s resulted.

In comparison with other works on the QDSSCs based on ZnO photoanodes, the results obtained in this work is quite competitive (S1 Table), confirming the effectiveness of utilizing hierarchical nanostructures in improving the QDSSC performance.

At a ZnO/CdS/electrolyte interface, some of the photoexcited electrons would recombine with the holes around the interface instead of moving forward into the photoanode. This recombination process would lower the *V*_*OC*_ of the cell [80]. Interestingly, although the employment of the hierarchical nanostructures increased the specific area of the photoanode, both the *V*_*OC*_ and *FF* of the ZNC/ZNS-based cell were still higher than that of the ZNC-based cell, though only slightly.

As shown in Fig 1C, the ZnO nanospikes provided more channels for the fast transportation of the photoexcited electrons into the photoanodes, thus these photoexcited electrons had a smaller probability of recombining with holes, giving rise to higher *V*_*OC*_ and *FF*. This slight increase in the *V*_*OC*_ and *FF* is echoed in the EIS results shown in Fig 5B and Table 2.

Theoretically, the Nyquist plot of a solar cell should contain three semicircles at low, medium and high frequencies [81,82]. The experimental data can be fitted with an equivalent circuit shown in the inset of Fig 5B. *R*_*1*_ in the equivalent circuit, which can be calculated by analyzing the low frequency part of the Nyquist plot, is a reflection of the Nerst diffusion of the I^-^and I_3_^-^ anions in the electrolyte [81]. *R*_*2*_ and *C*_*2*_ are the incarnations of the electrochemical reaction impedance at the Pt counter electrode and can be worked out from the left semicircles at high frequencies. *R*_*3*_ and *C*_*3*_, whose value can be estimated using the semicircle at the medium frequencies, are used to represent the charge transfer impedance at the QD/electrolyte/ZnO triple junction. All the recombination events between the electrons and holes in the ZnO photoanode, the electrolyte, and the QDs, which hindered the electron transfer from the QDs to the FTO substrate through the ZnO photoanode, contributed to *R*_3_ [30,83,84].

In the Nyquist plots shown in Fig 5B, only the semicircles at the medium frequencies are large enough for analysis, thus only the values of *R*_*3*_ and *C*_*3*_ are calculated by fitting. Fig 5B and Table 2 demonstrate that the resistance per unit area across the ZNC(3)/ZNS photoanode was slightly larger than that of the ZNC(3) photoanode (0.24 vs. 0.22 kΩ·cm^−2^), suggesting that the employment of the hierarchical nanostructures also reduced the recombination events, despite of an increase in the actual interface area. As a result, the lifetime of the photoexcited electrons at the interface, i.e., the multiplication of the resistance and capacitance, of the ZNC(3)/ZNS photoanode is longer than that of the ZNC(3) photoanode (1.4 vs. 0.9 ms). As previously reported, photoexcited electrons might diffuse to the FTO via the pathway of the secondary nanospikes and circumvented the primary nanorods [34,44]. On a ZNC/ZNS photoanode, a photoexcited electron had two possible pathways to the external circuit. Firstly, it could entered a ZNS first and then entered the underlying ZNC through the ZNC/ZNS interface. Secondly, it could move along the ZNS surfaces all the way to the FTO substrate. The probability of a photoexcited electron recombining with a hole is high at the ZNC/ZNS interface. The fact that the lifetime of a photoexcited electron in the ZNC/ZNS photoanode was longer than that in the ZNC photoanode (1.4 vs. 0.9 ms, Table 2) suggests that most photoexcited electrons followed the conduction channels along the ZNS surfaces. The enlargement of surface area of the ZNC/ZNS photoanode provided more opportunities for the photoexcited electrons to diffuse to the external circuit.

The IPCE spectra in Fig 5C show that the employment of the hierarchical nanostructures did not change the shape of the response spectrum of the cell. Instead, it only greatly raised the response at the wavelengths between 0.4 to 0.5 μm.

## Conclusion

Applying hierarchical nanostructures, which can be obtained using a facile and inexpensive hydrothermal method, is an effective approach to improving the performance of a QDSSC based on a ZnO photoanode. The results of comparative experiments disclosed that the *J*_*SC*_, *V*_*OC*_ and *FF* were all raised when a photoanode of ZnO nanocones was replaced by a photoanode of ZnO nanocone/nanospike hierarchical nanostructures. As a result, a more than doubled conversion efficiency was attained. This improvement in the photovoltaic performances indicates that the employment of properly tailored hierarchical nanostructures can not only enhance the capability of a photoanode to load QDs and trap incident light, but also, to a lesser extent, to contain the recombination of the photoexcited electrons.

## Supporting Information

S1 Fig Longer growth time. (DOC)

S2 Fig Reflectance spectra and transmittance spectra. (DOC)

S3 Fig 20 samples. (DOC)

S1 Table Comparison. (DOC)
