# Supplementary material for: ZnO Hierarchical Nanostructure Photoanode in a CdS Quantum Dot-Sensitized Solar Cell
Source: PLoS One. 2015 Sep 17;10(9):e0138298. doi: 10.1371/journal.pone.0138298 (PMC4574909; doi:10.1371/journal.pone.0138298)
Supplement: S1 Fig — (DOC) [file pone.0138298.s001.doc]

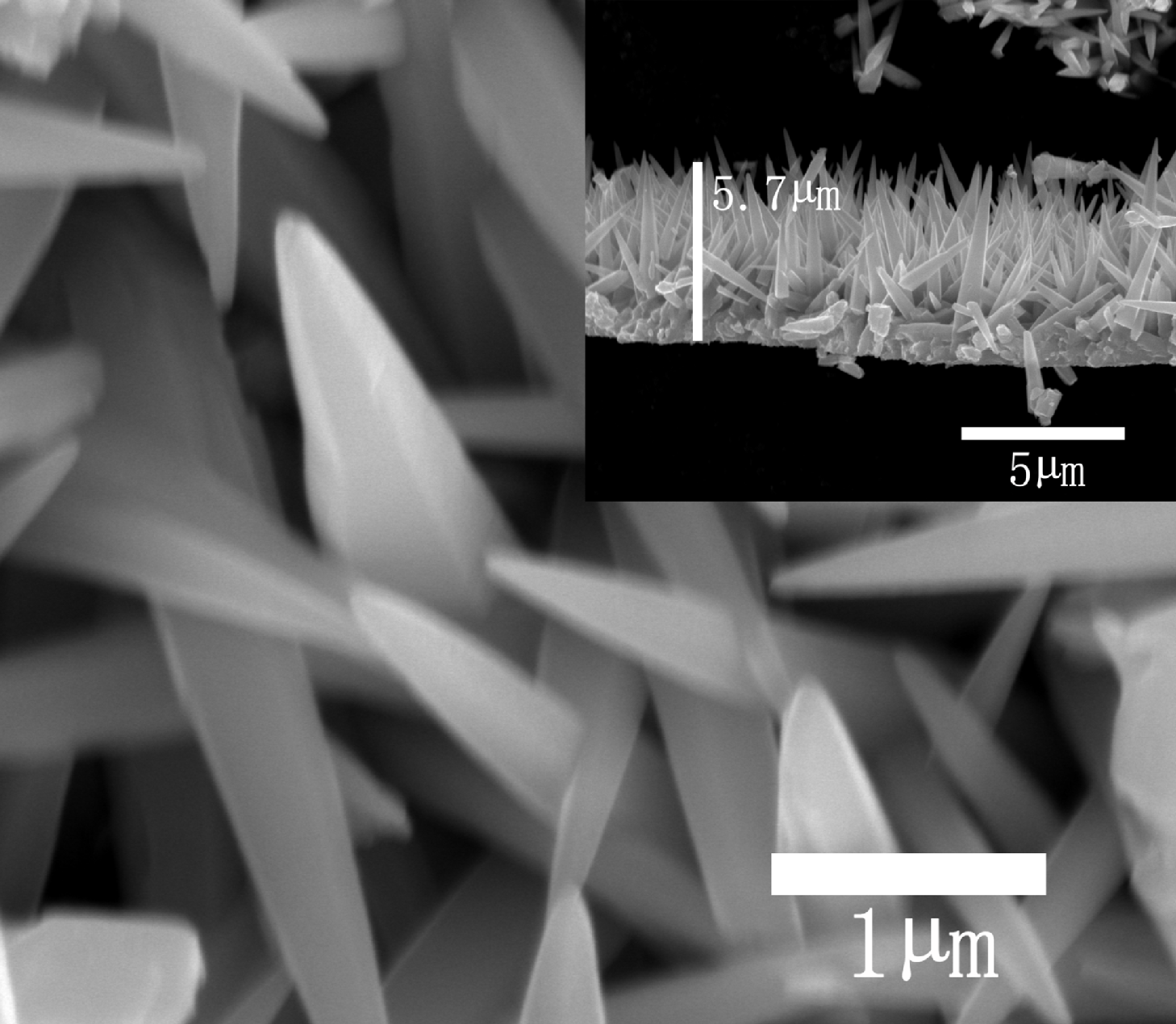

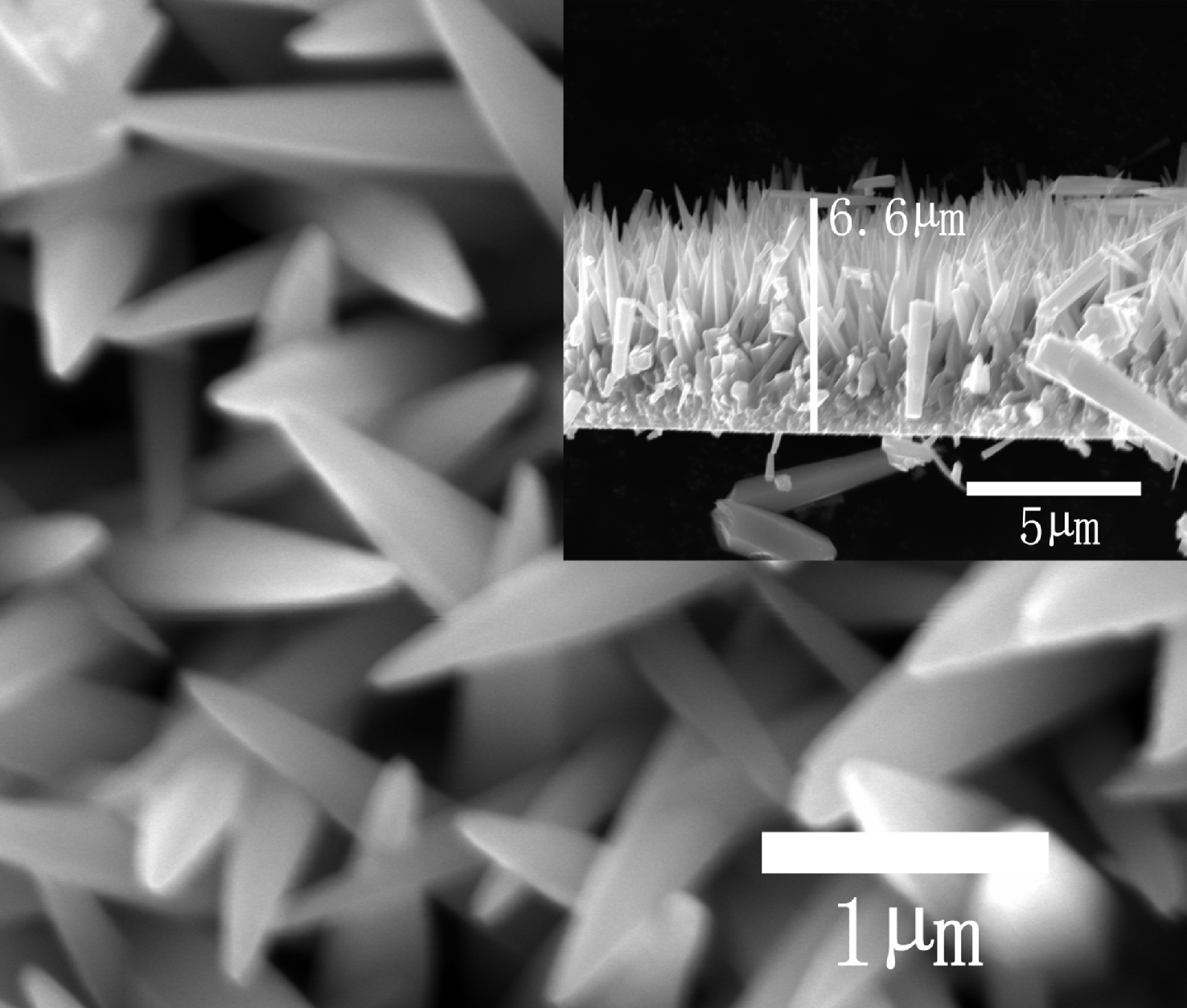


(a) (b)


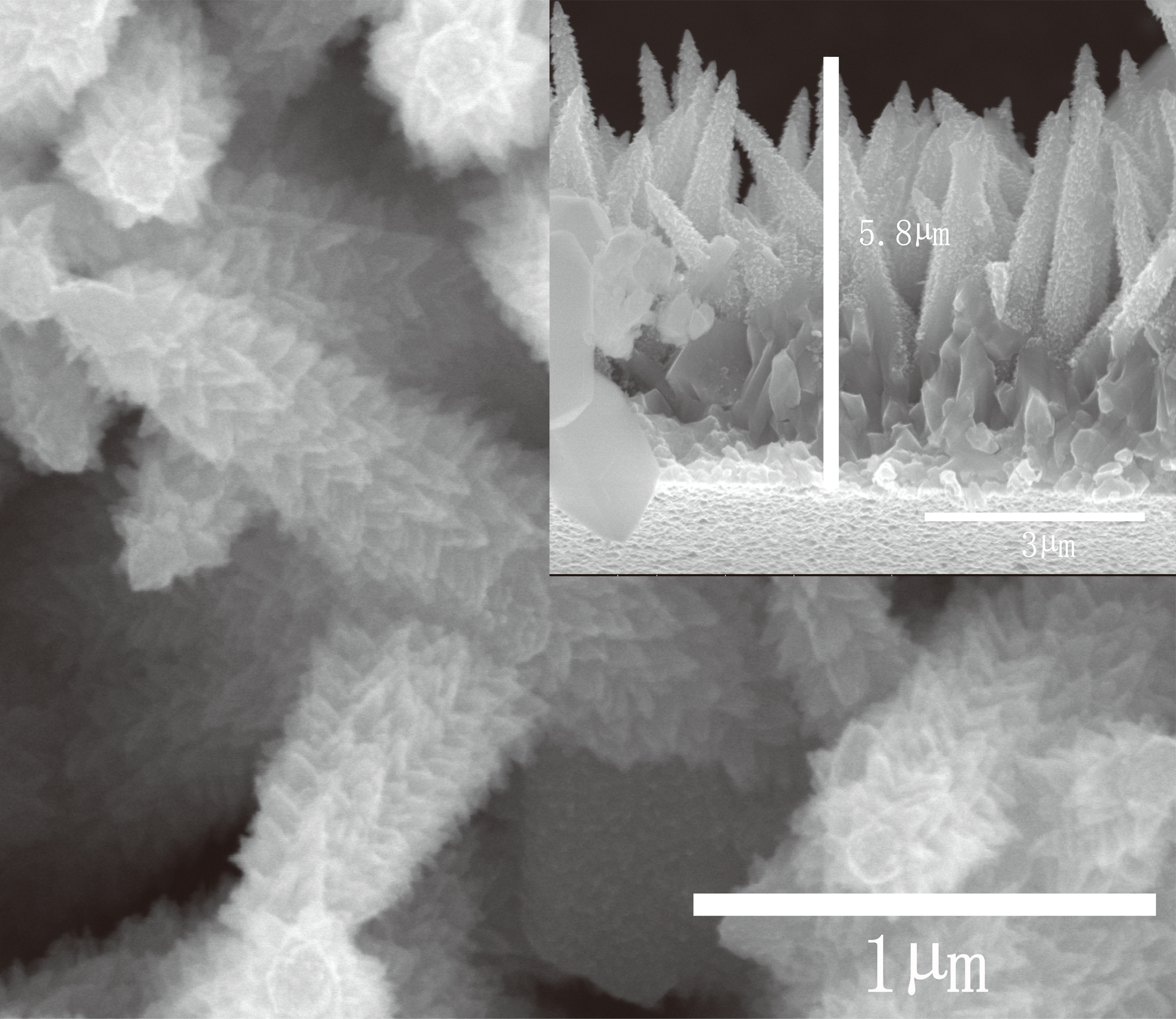

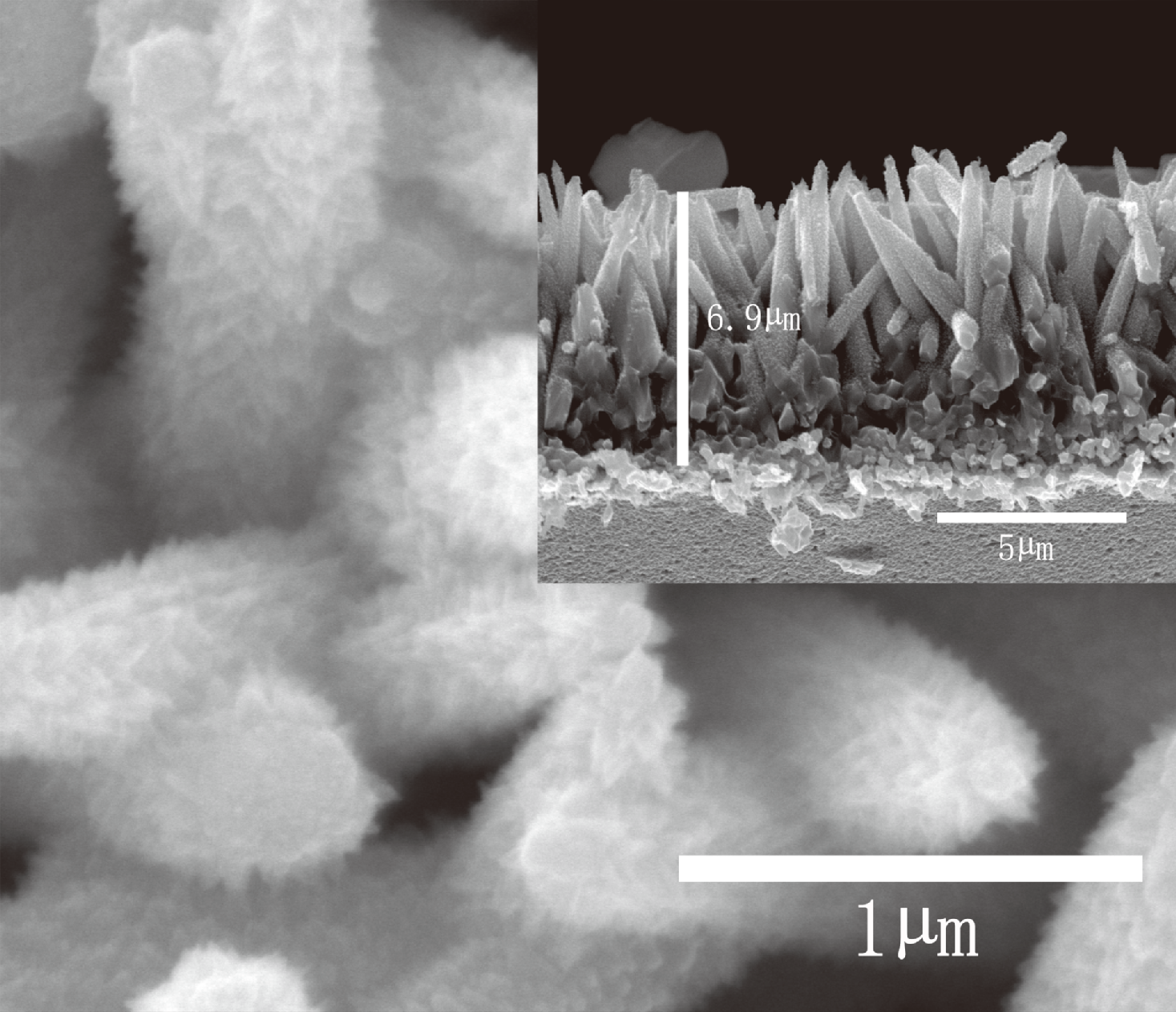


(c) (d)

**Figure S1** SEM images of the ZNC arrays and the hierarchical ZNC/ZNS arrays with longer growth time. (a) a ZNC(6) array, (b) a ZNC(9) array, (c) a ZNC(6)/ZNS array, and (d) a ZNC(9)/ZNS array.
