# Supplementary material for: ZnO Hierarchical Nanostructure Photoanode in a CdS Quantum Dot-Sensitized Solar Cell
Source: PLoS One. 2015 Sep 17;10(9):e0138298. doi: 10.1371/journal.pone.0138298 (PMC4574909; doi:10.1371/journal.pone.0138298)
Supplement: S2 Fig — (DOC) [file pone.0138298.s002.doc]

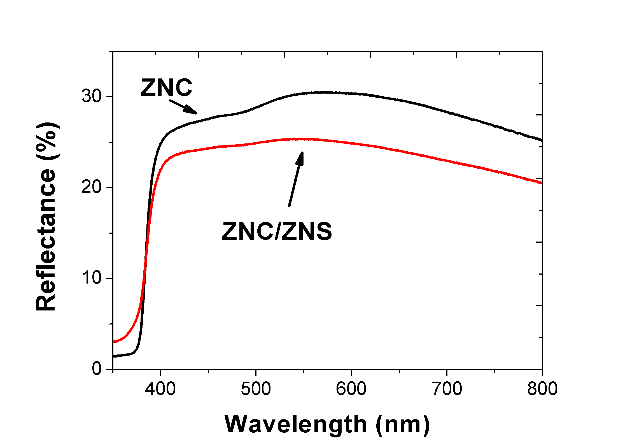

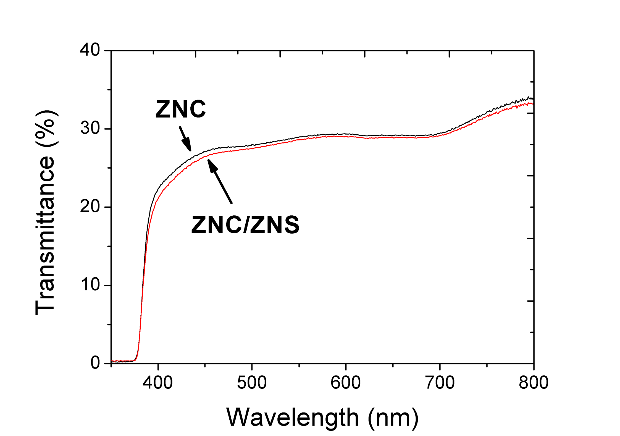


**(a) (b)**

**Figure S2** Reflectance spectra and transmittance spectra of the ZNC and ZNC/ZNS photoanodes.

(a) reflectance spectra and (b) transmittance spectra.
