# Supplementary figures and images for: ZnO Hierarchical Nanostructure Photoanode in a CdS Quantum Dot-Sensitized Solar Cell
Source: PLoS One. 2015 Sep 17;10(9):e0138298. doi: 10.1371/journal.pone.0138298 (PMC4574909; doi:10.1371/journal.pone.0138298)

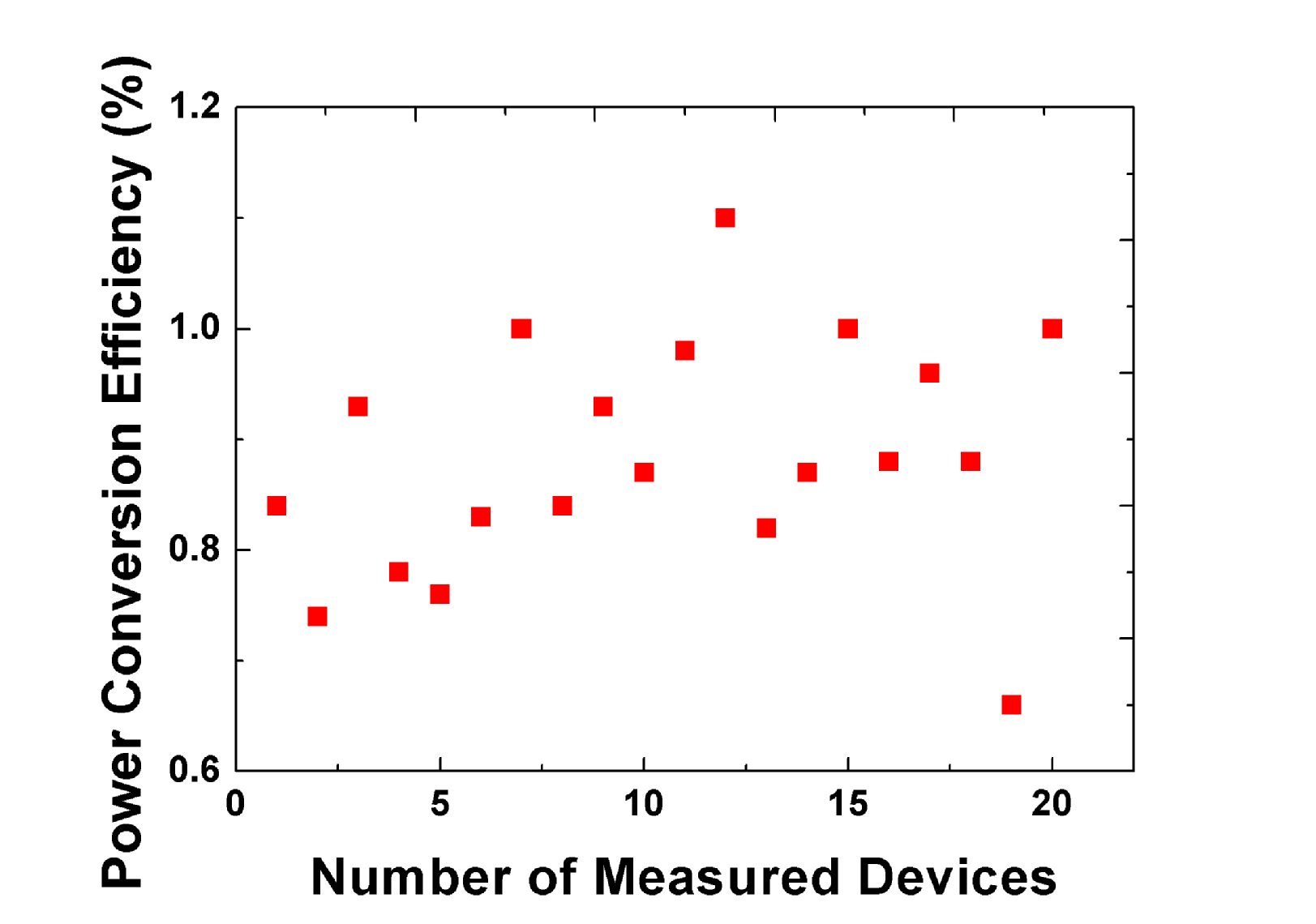


**Figure S3** Conversion efficiencies of 20 ZNC(3)/ZNS-based QDSSCs.

Supplement: S3 Fig — (DOC) [file pone.0138298.s003.doc]
