# Supplementary material for: ZnO Hierarchical Nanostructure Photoanode in a CdS Quantum Dot-Sensitized Solar Cell
Source: PLoS One. 2015 Sep 17;10(9):e0138298. doi: 10.1371/journal.pone.0138298 (PMC4574909; doi:10.1371/journal.pone.0138298)
Supplement: S1 Table — (DOC) [file pone.0138298.s004.doc]

**Table S1** QDSSCs based on ZnO photoanodes: a comparison

Photoanode (Ref.) sensitizer counter electrode η (%) electrolyte

Bilayer ZnO [1] CdSe Pt 0.45 Liquid

ZnO NT [2] CdS Pt 0.87 Liquid

CL-ZnO [3] CdS/CdSe Au 0.55 Liquid

ZnO NF [4] CdS Au 0.64 Solid

Zn_2_SnO_4_−ZnO [5] CdS/CdSe Pt 1.68 Liquid

ZnO–TiO_2_ NR [6] CdS Pt 0.73 Liquid

ZnO/AZO NCA [7] CdS Pt 0.88 Liquid

ZNC/ZNS (this work) CdS Pt 1.4 Liquid

Bilayer: nanorod/nanoflower, NT: nanotube, CL: nanowire array/nanoparticles, NF: nanofiber, NR: nanorods, AZO: Al-doped ZnO, NCA: nanocables

**References**

1. Chen J, Li C, Song JL, Sun XW, Lei W, Deng WQ. Bilayer ZnO nanostructure fabricated by chemical bath and its application in quantum dot sensitized solar cell. Applied Surface Science. 2009;255: 7508–7511. doi:10.1016/j.apsusc.2009.03.091

2. Yang L, Zhang Z, Yang J, Yan Y, Sun Y, Cao J, et al. Effect of tube depth on the photovoltaic performance of CdS quantum dots sensitized ZnO nanotubes solar cells. Journal of Alloys and Compounds. 2012;543: 58–64. doi:10.1016/j.jallcom.2012.07.057

3. Chou C-Y, Li C-T, Lee C-P, Lin L-Y, Yeh M-H, Vittal R, et al. ZnO nanowire/nanoparticles composite films for the photoanodes of quantum dot-sensitized solar cells. Electrochimica Acta. 2013;88: 35–43. doi:10.1016/j.electacta.2012.09.121

4. Wu S, Li J, Lo S-C, Tai Q, Yan F. Enhanced performance of hybrid solar cells based on ordered electrospun ZnO nanofibers modified with CdS on the surface. Organic Electronics. 2012;13: 1569–1575. doi:10.1016/j.orgel.2012.04.018

5. Li L-B, Wang Y-F, Rao H-S, Wu W-Q, Li K-N, Su C-Y, et al. Hierarchical Macroporous Zn _2_ SnO _4_ –ZnO Nanorod Composite Photoelectrodes for Efficient CdS/CdSe Quantum Dot Co-Sensitized Solar Cells. ACS Applied Materials & Interfaces. 2013;5: 11865–11871. doi:10.1021/am4035653

6. Liu B, Sun Y, Wang D, Wang L, Zhang L, Zhang X, et al. Construction of a branched ZnO–TiO2 nanorod array heterostructure for enhancing the photovoltaic properties in quantum dot-sensitized solar cells. RSC Adv. 2014;4: 32773–32780. doi:10.1039/C4RA05736H

7. Deng J, Wang M, Liu J, Song X, Yang Z. Arrays of ZnO/AZO (Al-doped ZnO) nanocables: A higher open circuit voltage and remarkable improvement of efficiency for CdS-sensitized solar cells. Journal of Colloid and Interface Science. 2014;418: 277–282. doi:10.1016/j.jcis.2013.11.017
